# Lung Transplantation for Pulmonary Arterial Hypertension: Optimized Referral and Listing Based on an Evolving Disease Concept

**DOI:** 10.3390/jcdd10080350

**Published:** 2023-08-15

**Authors:** Xiaokun Hu, Ningying Ding, Wanqiu Songchen, Ruifeng Wang, Jing Chen, Ailing Zhong, Jinzhu Nan, Yujie Zuo, Heng Huang, Dong Tian

**Affiliations:** 1 Outpatient Department, West China Hospital, Sichuan University, Chengdu 610041, China; huxiaokun@wchscu.cn; 2 Anesthesia Operation Center of West China Hospital/West China School of Nursing, Sichuan University, Chengdu 610041, China; dingningying@wchscu.cn; 3 Heart and Lung Transplantation Research Laboratory, North Sichuan Medical College, Nanchong 637000, China; songcwq_eatts@stu.nsmc.edu.cn (W.S.); wangruifeng_eatts@stu.nsmc.edu.cn (R.W.); chen-ching@outlook.com (J.C.); zhongal_eatts@stu.nsmc.edu.cn (A.Z.); nanjz_eatts@stu.nsmc.edu.cn (J.N.); zuoyj_eatts@stu.nsmc.edu.cn (Y.Z.); 4 Department of Thoracic Surgery, West China Hospital, Sichuan University, Chengdu 610041, China; 5 Lung Transplant Research Laboratory, Institute of Thoracic Oncology, West China Hospital, Sichuan University, Chengdu 610041, China

**Keywords:** pulmonary arterial hypertension, lung transplantation, referral, waiting list, risk stratification, lung allocation score

## Abstract

Pulmonary hypertension (PH) was once a devastating and fatal disease entity, the outlook of which has been significantly improved by the continued progress of medical treatment algorithms. However, some patients still ultimately fail to achieve an adequate clinical response despite receiving maximal medical treatment. Historically, lung transplantation (LTx) has been the only effective therapeutic option that could lead to satisfactory outcomes and save these advanced patients’ lives. However, patients with PH tend to have the highest mortality rates on the transplant waiting list; especially after comprehensive medical treatment, they continue to deteriorate very rapidly, eventually missing optimal transplantation windows. Balancing optimized medical treatment with the appropriate timing of referral and listing has been highly controversial in LTx for patients with PH. The 2021 consensus document for the selection of lung transplant candidates from the International Society for Heart and Lung Transplantation (ISHLT) updated the specific recommendations for the LTx referral and listing time for patients with PH based on objective risk stratification. Herein, we review the evolving PH-related concepts and highlight the optimization of LTx referral and listing for patients with PH, as well as their management on the waiting list.

## 1. Introduction

Pulmonary hypertension (PH) is a devastating disease process with complex pathogenesis and multi-factor interaction, which is characterized by progressive pulmonary vascular resistance, and ultimately leads to right ventricular failure and premature death [1,2]. PH affects approximately 1% of the global population, with the estimated incidence and prevalence higher in economically developed countries, ranging from 1.5 to 32 cases and 12.4 to 268 cases per million adults, respectively [3,4]. Over the past two decades, there has been a sea change in the understanding of the pathological mechanisms, diagnosis, and treatment algorithms of PH. Intravenous epoprostenol was a milestone in the medical treatment of patients with PH, which has been transformed into individualized multi-pathway combination medication strategies based on risk stratification [1,5]. Although the introduction of various targeted therapies has resulted in significant advances in the treatment of PH, none of the currently approved medical therapies are considered to be a definite cure, and the 5-year mortality rate remains high at about 20–60% [6]. In real-world clinical practice, some patients fail to achieve an adequate clinical response despite receiving maximal medical treatment. At this time, lung transplantation (LTx) is the ultimate therapeutic option to save the lives of patients with severe PH [7].

Decisions regarding the referral and listing time for LTx have been challenging in patients with PH since the disease course of PH is highly heterogeneous and unpredictable [8]. It has been reported that patients with PH tend to have the highest mortality rate on LTx waiting lists, ranging from 18% to 41%. In addition to the inherent severity of the disease, another noteworthy reason lay in that many patients deteriorated very rapidly after prolonged medical treatment, and thus patients with severe PH were prone to miss the optimal referral and transplantation window on account of delayed referral and listing, as well as a shortage of donor organs [2,9,10]. Therefore, the contradiction between adequate medical treatment and the appropriate timing of LTx referral and listing is currently a highly concerning and highly controversial topic in the PH field. In addition, concepts related to PH are changing constantly, which makes individualized treatment algorithms even more confusing. The 2021 consensus document for the selection of lung transplant candidates from the International Society for Heart and Lung Transplantation (ISHLT) has introduced fresh recommendations for LTx for patients with PH based on objective risk stratification, emphasizing the significance of early referral and listing [11]. This article will review the evolving PH-related concepts and highlight the optimization of LTx referral and listing for patients with PH, aiming to contribute to facilitating successful LTx and improving the survival outcomes of patients with severe PH.

## 2. Evolving Disease Concepts Associated with PH

### 2.1. Hemodynamic Definition and Classification of PH

Since the first World Symposium on Pulmonary Hypertension (WSPH) in 1973, PH has been defined as mean pulmonary arterial pressure (mPAP) ≥ 25 mmHg at rest, measured by right heart catheterization in the supine position [12]. With the deepening of PH clinical and translational medicine research, it has been recognized that this upper limit of normal mPAP of 25 mmHg was somewhat empirical and arbitrarily defined [13]. Until 2018, the sixth WSPH redefined PH as mPAP > 20 mmHg at rest and added the parameter of pulmonary vascular resistance (PVR) based on the pulmonary arterial wedge pressure (PAWP) to better distinguish between pre-capillary PH and post-capillary PH [12]. Based on the 2015 ESC/ERS guidelines for the diagnosis and treatment of PH (ESC/ERS PH Guidelines) and the sixth WSPH, the latest 2022 ESC/ERS PH Guidelines adopted this new standard and introduced a transformative update of PH-related concepts [1,12,14]. The new clinical classifications of PH are as follows: Group 1—pulmonary arterial hypertension (PAH); Group 2—PH associated with left heart disease; Group 3—PH associated with lung diseases and/or hypoxia; Group 4—PH associated with pulmonary artery obstructions; and Group 5—PH with unclear and/or multifactorial mechanisms. Of these, PAH (Group 1), the narrative object group of this review, is the focus of current clinical practice [1,15].

### 2.2. Risk Stratification of Patients with PAH

Over the decades, various calculators have been designed to distinguish the severity and predict outcomes of patients with PAH [14,16,17,18,19,20,21]. The US Registry to Evaluate Early and Long-term PAH Disease Management risk calculator (REVEAL risk calculator), and the ESC/ERS PH Guidelines risk table are two of the most commonly used risk stratification tools. The original REVEAL risk calculator was an algorithm that used 12 baseline parameters to calculate the risk score, predicting 12-month survival and providing useful serial survival assessments for patients with PAH [19,22,23]. REVEAL 2.0 risk calculator was a modification of the original REVEAL risk calculator, incorporating a new variable (all-cause hospitalization) and a revised variable (renal function measured by estimated glomerular filtration rate) [24]. However, the limitations of the commonly used REVEAL risk calculator include the relatively short prediction period (1 year) and the large number of variables required (from 12 to 14 variables) at follow-up assessment. A previous study showed that simplified versions of the REVEAL scores utilizing high-yield variables appeared to have comparable performance to the original REVEAL risk calculator, which requires more in-depth data to validate [23].

In addition, PAH patients can be classified as low-risk, intermediate-risk, or high-risk for clinical worsening or death using the 2015 ESC/ERS PH Guidelines risk table [14]. The main evaluation parameters include clinical signs of right heart failure, syncope, World Health Organization (WHO) functional class, six-minute walk test (6 MWT), cardiopulmonary exercise testing, *N*-terminal prohormone of brain natriuretic peptide (NT-proBNP) plasma levels, imaging (echocardiography and cardiac magnetic resonance imaging (cMRI)), and hemodynamics [14]. It was reported that, based on 12-month mortality, the correspondence between the low-, intermediate-, and high-risk groups as defined by the 2015 ESC/ERS PH Guidelines and the REVEAL 2.0 risk calculator (14 variables) was as follows: low-risk = REVEAL score ≥ 6; intermediate-risk = REVEAL score 7 and 8’ and high-risk = REVEAL score ≥ 9 [21,24].

To deal with the fact that 1-year mortality was sometimes higher than predicted in patients with moderate–high risk PAH and that 60–70% of patients are classified as intermediate risk, the latest 2022 ESC/ERS PH Guidelines presented a transformative improvement in the methods and parameters for risk stratification in patients with PAH [1,18,20]. For risk stratification at initial diagnosis, a three-strata model (based on estimated 1-year mortality rates of <5%, 5–20%, and >20%, respectively) will be recommended, with as many variables included as possible for a comprehensive assessment; while at follow-up, the simplified four-strata model will be preferred, classifying patients into low-risk, intermediate–low-risk, intermediate–high-risk, and high-risk [1]. It should be noted that the three-strata model further expanded the risk stratification factors to include echocardiography, cMRI, and some others, which would be a more accurate and scientific assessment, but these indicators were difficult to obtain in clinical practice.

Collectively, the available research supported a risk-based approach that ultimately enabled patients with PAH to achieve and/or maintain a low-risk status, or to consider the timing of the referral and listing for a potential LTx [25,26,27]. It is worth mentioning that the current risk stratification tools are not perfect and will be gradually optimized with the deepening of the understanding of PAH. Therefore, the combined use of multiple risk stratification tools may provide more information on survival prediction for patients with PAH [11].

## 3. Treatment Algorithms for Patients with PAH

With the clinical application of vasodilatory drugs, the survival time of patients with PAH has increased from 2.8 years in the 1980s to more than 7 years in the modern era [28]. The main clinical treatment drugs include prostacyclin, endothelin receptor antagonists (ETRAs), phosphodiesterase 5 inhibitors (PDE5is), soluble guanylate cyclase stimulators (sGCs), and so forth [15,29,30,31,32,33,34,35,36,37]. However, there have been no definitely effective drugs to reverse or cure the PAH progression [38]. The contribution of LTx to saving the lives of patients whose condition is difficult to optimize with medical treatment is the focus of this review.

Organ transplantation, including heart–lung transplantation (HLTx) and LTx for PAH, is a young field, the main principle of which is that transplant programs should be positively considered when patients with PAH continue to deteriorate despite receiving optimized medical treatment [2,9]. In 1963, James Hardy and colleagues performed the first human LTx. Unfortunately, the patient survived only eight days postoperatively [39]. There was no significant progress in LTx for the next 15 years, until the 1980s when cyclosporine was first applied as an immunosuppressive therapy, a major milestone in LTx history. In 1981, Stanford University performed the first successful HLTx on a patient with “primary pulmonary hypertension” and incorporated the concept of HLTx/LTx into clinical practice for severe PAH [40]. Shortly thereafter, with the first long-term successful single LTx and significant advances in bilateral sequential LTx, the selection of LTx has become recognized as an effective treatment for PAH [9,11,41,42].

**Figure 1:**
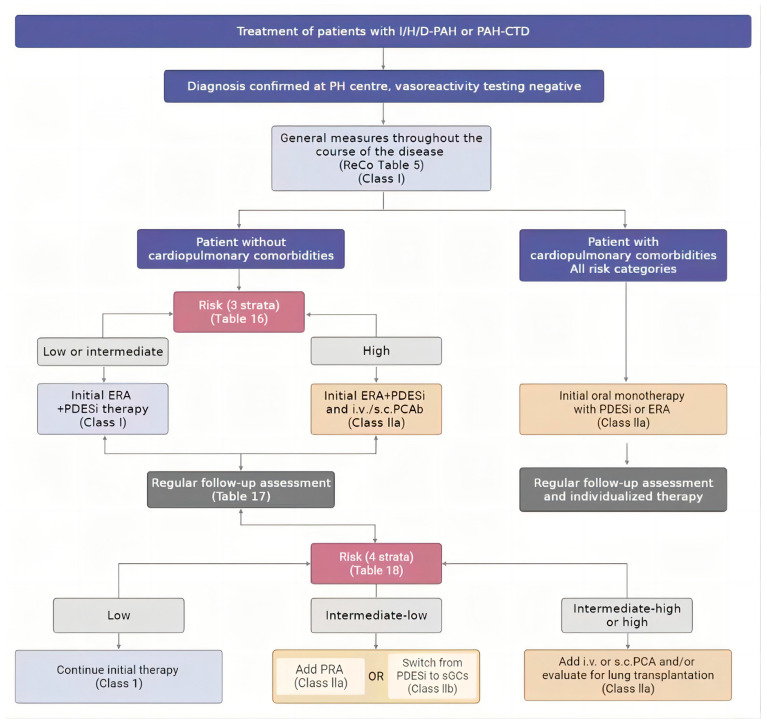
Pulmonary arterial hypertension treatment algorithms for patients with idiopathic, heritable, drug-associated, and connective-tissue-disease-associated pulmonary arterial hypertension [1]. DLCO, lung diffusion capacity for carbon monoxide; ERA, endothelin receptor antagonist; I/H/D-PAH, idiopathic, heritable, or drug-associated pulmonary arterial hypertension; i.v., intravenous; PAH-CTD, PAH associated with connective tissue disease; PCA, prostacyclin analog; PDE5i, phosphodiesterase 5 inhibitor; PH, pulmonary hypertension; PRA, prostacyclin receptor agonist; ReCo, recommendation; s.c., subcutaneous; sGCs, soluble guanylate cyclase stimulators. Cardiopulmonary comorbidities are conditions associated with an increased risk of left ventricular diastolic dysfunction, and include obesity, hypertension, diabetes mellitus, and coronary heart disease; pulmonary comorbidities may include signs of mild parenchymal lung disease and are often associated with a low DLCO (<45% of the predicted value). b Intravenous epoprostenol or i.v./s.c. treprostinil.

Formerly, HLTx and single LTx were the preferred treatments for patients with PAH. However, it is currently more popular for patients with PAH to receive bilateral LTx in most cases [43,44,45,46,47]. Impaired ventilation–perfusion mismatching may occur after a single LTx, which may lead to subsequent rejection, infection, primary graft dysfunction (PGD), and even severe hypoxia or early death after surgery (20% mortality risk within 1 month). Additionally, potential right ventricular dysfunction and high pulmonary blood flow after a single LTx can increase the incidence and severity of PGD [48]. In a single-center experience, the 5-year survival rate of recipients with PAH who underwent bilateral LTx and single LTx reached 84% and 51%, respectively, which further clarified the survival benefit of bilateral LTx [49]. Moreover, a severe shortage of heart–lung blocks has been a significant obstacle in the field of HLTx, and henceforth HLTx will be reserved for patients with other uncorrectable heart conditions, including Eisenmenger syndrome complicating complex congenital heart disease (CHD), failed CHD repair, uncorrectable CHD, and severe left ventricular failure [50]. Another reason for this transformation is the growing understanding that the primary pathophysiological variation of PAH is in the pulmonary vessels, rather than inherent right ventricular problems [43]. Actually, studies have shown that pressure-overloaded right ventricles can be remodeled and recovered after bilateral LTx [2,51,52]. Previously, we reviewed 10 studies in end-stage cardiopulmonary disease, which involved 1230 patients with bilateral LTx and 1022 patients with HLTx. The results showed that 1-year, 3-year, 5-year, and 10-year survival rates were comparable between the two groups [53]. Also, bilateral LTx has the advantages of shorter waiting times and greater donor allocation. Since the 2010s, consensus statements for the selection of lung transplant candidates have supported bilateral LTx as the primary surgical treatment for severe PAH [54] (Table 1, Figure 1).

## 4. LTx Referral Time for Patients with PAH

### 4.1. Dilemmas of Transplant Referral

In the golden age of LTx, the selection of LTx for patients with PAH remained much rarer than for other diseases [2]. In the early stages, LTx for patients with PAH accounted for 13% in 1990 and 2% in 2007 [55]. From January 1995 to June 2018, only 1863 cases of idiopathic PAH and 978 cases of other causes of PH were registered in ISHLT, accounting for only 2.9% and 1.5% of all transplant cases in the same period, respectively [56]. The question is, where are the patients with PAH who are supposed to receive LTx?

We should be aware that LTx may still be an underutilized treatment for PAH [2]. Primarily, some patients may voluntarily forgo the LTx program for any number of reasons, whether religious beliefs or financial pressures. There are also some PAH patients with existing contraindications to transplantation, who are unable to derive a higher survival benefit from LTx. However, a more important consideration is the trade-off between adequate medical treatment and early referral for LTx in clinical practice [8,14,57]. Specifically, since the introduction of targeted therapies such as epoprostenol, there have been significant improvements in hemodynamics, exercise capacity, and 5-year survival in patients with PAH [5,58,59]. However, the disease course of PAH is unpredictable, and many patients receive effective medical treatment from the time of diagnosis, which results in a refusal of the LTx program. As the disease progresses, many patients become insensitive to medication and even continue to deteriorate rapidly [60]. When patients with PAH are referred at the start of clinical deterioration, there may not be enough time to finish the assessment and obtain a suitable donor organ, and there is even a risk of death on the waiting list. Due to the expectation of medical treatment, physicians may fall into the “trap” of optimized medical treatment, neglecting early referrals for LTx in the course of PAH and ending with the patient missing the transplantation window [2,61]. These conditions may be more troublesome in physicians coming from non-PH centers. Given the complexity of PAH management, physicians in primary-care centers should conduct a comprehensive assessment of patients with PAH, starting by predicting the disease progression according to the patient’s physical condition and risk factors, and formulating reasonable medical treatment strategies. Depending on the effectiveness of the medical intervention, patients could be referred to a superior medical facility with a PH center for the foreseeable future.

### 4.2. Referral Time Strategies

In order to resolve the dilemma of LTx referral or even listing in patient populations with PAH, the 2014 ISHLT consensus document recommended referral for LTx when advanced symptoms or rapidly progressive disease were present despite an escalation of therapy [54]. It is not hard to recognize that a previous LTx program is the “ultimate lifeline” after failed medical treatment. However, our brief is that “ultimate lifeline” does not equate to “only decide at the last minute”, that is, an LTx referral for patients with PAH should be performed as early as possible to cope with deteriorating conditions [11,14,21,62].

**Figure 2:**
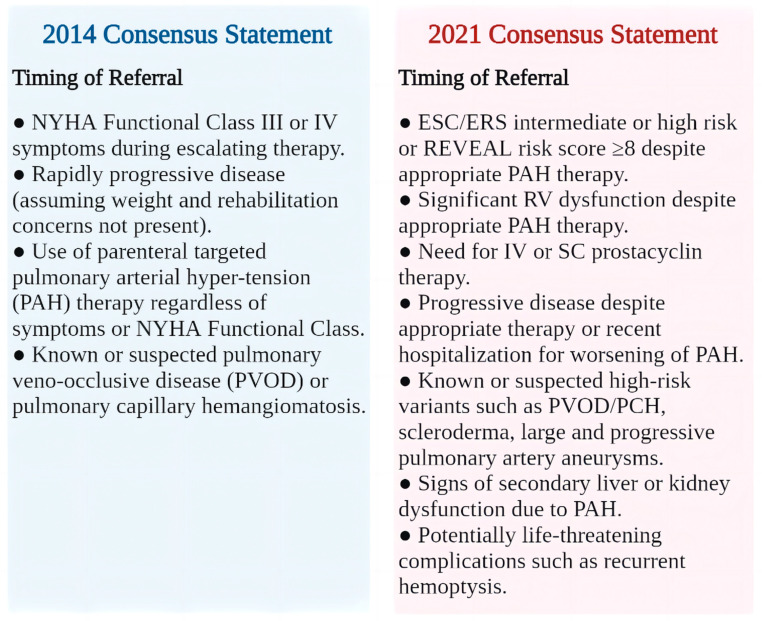
Lung transplantation referral time for patients with pulmonary arterial hypertension [11]. NYHA, New York Heart Association; ESC/ERS, European Society of Cardiology/European Respiratory Society; REVEAL, Registry to Evaluate Early and Long-term PAH Disease Management; RV, right ventricular; IV, intravenous; SC, subcutaneous; PCH, pulmonary capillary hemangiomatosis.

In the era of risk-assessment-based treatment, risk stratification of patients with PAH is particularly significant. Under the previous treatment algorithms, physicians relied more on diagnosis and treatment procedures than objective risk stratification, which led to a serious underestimation of prognostic risk in those patients [2,21]. According to REVEAL risk calculator 2.0 and the 2015 ESC/ERS PH Guidelines risk table, the sixth WSPH in 2018 indicated that patients with PAH at all risk levels who failed to reach low-risk status after 3–6 months of initial treatment required intensive treatment. If low-risk status has not been achieved after 3–6 months of maximum treatment, then patients should be referred for LTx evaluation [21]. The 2021 ISHLT consensus document was introduced, the core content of which was that after adequate drug therapy, patients with intermediate or high risk based on the 2015 ESC/ERS PH Guidelines risk table or REVEAL risk score ≥ 8 should be considered for LTx referral [11]. Interestingly, the latest 2022 ESC/ERS PH Guidelines further revolutionized the 2015 ESC/ERS risk stratification model and also presented a new consensus on LTx referral timing for PAH. However, the applicability of this modified model needs to be validated by more prospective studies [1,21] (Figure 2).

Additional clinical data need to be considered during risk assessments, including cardiopulmonary exercise tests and right ventricular assessment by echocardiogram and/or cMRI, as well as specific clinical conditions known to be equivalent to high risks, such as renal dysfunction, liver dysfunction, pulmonary veno-occlusive disease/pulmonary capillary hemangiomatosis (PVOD/PCH), connective-tissue-disease-associated pulmonary arterial hypertension, etc. [1,2,63,64]. Some evidence suggests that morbidity and mortality from PAH are driven by the influence of pulmonary hemodynamic dysfunction on renal dysfunction. In a REVEAL registry analysis, a ≥10% decline in estimated glomerular filtration rate (eGFR) for more than 1 year was an independent predictor of poorer survival in patients with PAH [65,66]. The balance between nephrotoxic immunosuppressive drugs and renal function after LTx is also complex and needs to be carefully evaluated at transplant referral. In addition, the main circulatory disturbances affecting liver function are congestion from right heart failure and ischemic injury from a low-cardiac-output state, which can be a serious consequence of severe PAH [67]. Impaired synthetic liver function and decreased albumin levels were also shown in multiple studies to be strong independent predictors of poor outcomes in patients with PAH [68,69]. Although transplant referrals are too aggressive for moderate-risk patients, given that LTx presents unique challenges in patients with PAH, we should recognize the need for early referral in this population [38]. It is worth mentioning that patients such as PVOD that are known to respond poorly to medical treatment should be referred promptly [9,54]. Patients with POVD/PCH may have a higher risk of dying on the waiting list compared to patients with PAH in the era of lung allocation. An analysis from the United Network for Organ Sharing (UNOS) database showed that 22.6% of patients with PVOD died on the LTx waiting list, compared to 11% of patients with PAH at 6 months [70]. Therefore, given the limited options and the severity of this disease, an early referral for LTx is recommended as soon as POVD/PCH is diagnosed. In addition, it is recommended to closely monitor disease progression and apply for additional transplant priority for such patients when appropriate. The same is true for scleroderma, although LTx is currently rare in patients with scleroderma, which accounts for only 1.1% of all LTx [71]. There is concern that the complex and severe extra-pulmonary manifestations of scleroderma may further reduce survival after LTx. However, recent studies have shown that post-transplant survival and chronic allograft lung dysfunction in patients with scleroderma are comparable to patients with other indications [71]. Therefore, early referral for patients with scleroderma is also warranted [11].

### 4.3. Additional Benefits of Early Referral

For patients in the high-risk group, either during initial treatment or optimized treatment, medical treatment and referral for LTx evaluation could theoretically be performed simultaneously [2], which was reflected in the treatment algorithms from the sixth WSPH, but not covered by the latest 2022 ESC/ERS PH Guidelines [1,2,21]. We need to fully recognize that early referral does not mean early listing, but rather giving patients a tentative and comprehensive assessment, promoting transplant education, and addressing risk factors or potential causes of ineligibility for LTx in advance, such as obesity, infection, psychological problems, etc. Once the clinical condition deteriorates, the LTx listing can be performed in a timely manner [11,38,61,72]. In addition, an early referral may improve a patient’s chances of surviving to transplantation and reduce disease severity at the time of transplantation, and those whose physical condition worsens during the waiting period due to delayed referral and listing may have lower postoperative survival and quality of life even if the LTx is successful [38]. The proposal for early referral upended the notion of passive waiting on the waiting list, giving patients with PAH the opportunity to use the waiting time to proactively complete more transplant-related preparations.

## 5. LTx Listing Time for Patients with PAH

### 5.1. Listing Time

Referred patients with PAH will be formally evaluated for LTx eligibility, which involves routine indications and contraindications [11]. This is a multidisciplinary process that must consider the overall clinical conditions of patients with PAH, including disease severity, psychological state, compliance, etc., which also means striking a balance between “patients who are severe enough” and “patients who are still in time for LTx”. Thus, it was indirectly demonstrated that early referral was beneficial for patients to be given sufficient time for a full assessment [38,61].

**Figure 3:**
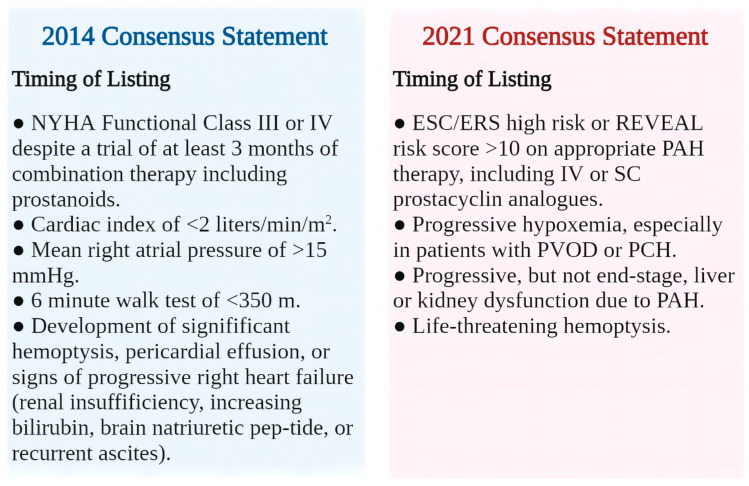
Lung transplantation listing time for patients with pulmonary arterial hypertension [11]. NYHA, New York Heart Association; ESC/ERS, European Society of Cardiology/European Respiratory Society; REVEAL, Registry to Evaluate Early and Long-term PAH Disease Management; RV, right ventricular; IV, intravenous; SC, subcutaneous; PCH, pulmonary capillary hemangiomatosis.

The risk stratification of patients with PAH is also tightly associated with the listing, and when patients show a high risk of short-term death, the listing should be considered despite optimized medical treatment [1,11]. Previously, LTx listing for patients with PAH was mostly based on the 2014 ISHLT consensus document, while the rapid development of risk stratification tools has brought more objectivity to this listing decision [9,54]. Based on these tools, the 2021 ISHLT consensus document has adjusted the listing criteria to the following: (1) ESC/ERS high-risk or REVEAL risk score >10 on appropriate PAH therapy, including IV or SC prostacyclin analogs; (2) progressive hypoxemia, especially in patients with PVOD or PCH; (3) progressive, but not end-stage, liver or kidney dysfunction due to PAH; and (4) life-threatening hemoptysis 24 (100%) [11]. As mentioned above, the risk stratification of patients with PAH saw a breakthrough in the 2022 ESC/ERS PH guidelines, but the translation of this breakthrough into clinical practice needs to be validated by more in-depth studies. It is clear that all current efforts toward risk stratification are aimed at enabling a more objective assessment of the severity of PAH to facilitate successful referral and listing. However, early referral does not mean blind early listing under any circumstances. Patients with PAH should not be actively placed on the waiting list until all other available treatment options have been exhausted, as LTx itself is also a high-risk procedure, so transplant surgeons need to prudently evaluate the risks and benefits of LTx [54] (Figure 3).

### 5.2. Allocation of Donor Lung

If patients with PAH meet the criteria and are on an active waiting list, there are policies in place to determine the patients’ priority relative to other candidates [7]. In the early days, this priority was mainly based on the cumulative time a patient spent on the waiting list, but it was unfavorable to patients whose condition deteriorated faster [72]. In 2005, the lung allocation score (LAS) was introduced to identify LTx priority according to the composite scores associated with the underlying disease severity, rate of clinical decline, and risk of death on the waiting list [73,74]. The LTx rates and waiting times for almost all end-stage lung diseases benefited from the LAS, effectively addressing the problem of the high mortality rate on the waiting list. However, the LTx rates for patients with PAH remain lower compared to other diagnoses, and mortality on PAH waiting lists is increasing, suggesting that LAS may be significantly underestimating the risk of death in patients with PAH [8,72,75,76]. In order to assess disease severity and mortality in PAH more accurately, various proposals led to a revision of the LAS algorithms in 2015, including reduced cardiac index and 6MWD, and increased resting oxygen consumption, creatinine, and bilirubin [8,38,77]. For those patients whose calculated LAS score may not adequately reflect the urgency of and expected outcome after LTx, some regions allowed for an exceptional LAS to be given higher priority [78,79].

In addition, some proposals for urgent LTx, such as the high-priority allocation protocol introduced in France, have also significantly reduced the mortality on waiting lists in patients with PH [80]. Specifically, urgent LTx is a prioritized allocation strategy for donor LTx according to the urgency of diseases, aiming to shorten the waiting time for donor lungs and reduce the fatality rate of patients on the waiting list for LTx [81]. However, the specific protocols and standards for urgent LTx are not completely unified, and the boundaries between urgent strategies and the classic LAS system are blurred. The urgent LTx quotas in various countries ranged from 8.3% to 28% [78,82,83,84]. Interestingly, transplant centers in Spain do not strictly limit the fixed quota of urgent LTxs whose indications only included invasive mechanical ventilation and severe PAH, and those patients with severe PAH who underwent an urgent LTx had a more optimistic prognosis trend [80]. The introduction of the concept of urgent LTx has provided new insights into LTx in patients with severe PAH, encouraging more attempts at allocation in this rapidly progressive disease.

Objectively, high waiting list mortality should not be entirely blamed on the allocation system, as some patients with PAH are actually in a very critical state before the listing, which also indirectly proves the importance of early referral [60]. Therefore, we should take an objective attitude to the listing of patients with PAH and evaluate it according to the severity and risk of the disease. Patients who deteriorate rapidly in a short period deserve more attention and priority. However, blind premature listing can conflict with transplant priorities, significantly lengthening waiting time and leading to an increase in unnecessary waiting list deaths. As mentioned above, advanced medication therapy can be performed at the same time as an LTx referral, which does not imply a definite possibility of listing. When patients with PAH become relatively stable during evaluation without obvious organ failure, or reversible factors and poor psychological adherence occur, physicians could terminate the LTx referral and listing and closely monitor the disease progression in order to reactivate the evaluation procedure if the conditions worsen.

## 6. Other Management on the Waiting List

When a patient with PAH is placed on the LTx waiting list, medical treatment for PAH ought to continue, aiming to prepare the patient for LTx and to perform at peak condition [21,38]. However, medical management is particularly challenging in severe PAH patients due to alterations in medication pharmacokinetics resulting from end-stage organ damage [85]. In addition to the prescribing of epoprostenol, volume management and positive inotropic drugs are equally important, given that the increased afterload in the pulmonary circulation leads to right ventricle remodeling and ultimately failure through various mechanisms [85,86]. Volume management in patients with PAH includes managing diuretic use and electrolyte imbalance, as well as monitoring fluid retention due to the use of endothelin receptor antagonists or calcium channel blockers [85]. Briefly, pre-transplant management is a multidisciplinary, collaborative procedure, so it is critical to establish close collaboration between PAH physicians and transplant surgeons.

In addition, for many eligible patients, bridging strategies may be required to keep patients alive while on the waiting list and avoid irreversible end-organ damage [87]. The risk of death from PAH is total heart failure due to progression of right heart failure, and cardiac arrest secondary to arrhythmia. In order to transition medical treatment to a successful LTx, extracorporeal membrane oxygenation (ECMO) can act as an important bridging strategy [2,88,89]. Previous studies have shown that ECMO can increase the survival rate and decrease the incidence of postoperative complications when compared to cardiopulmonary bypass (CPB) [90,91]. The specific strategies of ECMO for LTx in PAH patients should be developed by a multidisciplinary team of pulmonologists, intensivists, surgeons, and ECMO specialists. Even though venovenous ECMO (VV-ECMO) has multiple clinical advantages in terms of the operation and associated risk, a low risk of bleeding, and less anticoagulation, it is usually not suitable in PAH patients because VV-ECMO requires the patient’s right ventricle to function as the system’s pump [92]. Distinctively, in the presence of an atrial septal defect or a large patent foramen ovale, VV-ECMO can be inserted into the right internal jugular vein using a double-lumen catheter, so as to inject returned blood directly into the atrial septum to form an oxygenated right-to-left shunt [93]. However, as soon as heart function deteriorates in PAH patients, urgent conversion to VA-ECMO is required. VA-ECMO is a commonly used option that bypasses the original high-resistance pulmonary circulation, reduces the right ventricular load, and delivers oxygenated blood directly to the systemic circulation to improve end-stage organ function [8]. In a study of interstitial lung disease patients with PH, the survival of LTx recipients in initial VA-ECMO was significantly higher than that in VV-ECMO, with a 59% lower risk of death compared to VV-ECMO [94]. However, several serious complications limit the long-term use of ECMO, including hemolysis, intubation site bleeding, sepsis, cerebrovascular accidents, and multiple organ failure [72].

In addition, Fischer et al. introduced the application of a Novalung pumpless lung assist device with conduits (Novalung LAD), which can be considered as a bridge to LTx or HLT [95,96,97]. The Novalung device connects a low-resistance diffusion membrane parallel with the pulmonary artery to the left atrium (PA-LA). The inherent right ventricular cardiac output drives blood flow through a low-resistance diffusion membrane, where blood is oxygenated and returned to the systemic circulation without pumping [60,72]. Compared to VA-ECMO, PA-LA Novalung has allowed a reduction in inotrope support, an improvement in gas exchange parameters, optimization of ventilatory support requirements and extubation in selected cases, prolonged cardiopulmonary support, and recovery of right ventricular function [72].

For patients with PAH who cannot receive mechanical circulatory support to bridge to LTx, balloon atrial septostomy (BAS) or the Potts shunt could be considered [2,98,99]. BAS is a procedure that uses balloon dilation to create an atrial shunt through a right cardiac catheter to achieve right ventricular decompression and improve cardiac output by increasing the left ventricular preload [100]. However, the mortality associated with BAS is about 10%, and it is limited to symptomatic relief and emergency use [100]. In extremely rare cases, when ECMO and BAS are contraindicated, the Potts shunt may be considered to decompress the right ventricle and bridge the patient for transplantation [2,99].

## 7. Conclusions

LTx remains the ultimate therapeutic option for patients with PAH who have failed to respond to maximum medical treatment. The LTx referral and listing serve as an extremely essential first step to performing this salvage surgery successfully. In recent years, the definition, classification, and risk stratification of PAH, as well as comprehensive treatment algorithms, have made radical progress. Physicians and transplant surgeons should grasp this opportunity, continuously optimizing medical treatment algorithms, and dealing with LTx referral and listing by favoring early and objective assessments, respectively. For each patient with severe PAH, the risks and benefits must be prudently weighed to determine the most appropriate transplant strategy.

## Figures and Tables

**Table 1:** Advantages and disadvantages of single lung transplantation, bilateral lung transplantation, and heart–lung transplantation for pulmonary arterial hypertension.

Surgical Type	Advantages	Disadvantages
Single LTx	Less anesthesia, operative, and bypass time More accessible donor lungs More equitable and reasonable allocation of donor lungs	Poor survival compared to bilateral LTx and HLTx Infection risk for native lung Poor amelioration of pulmonary pressure Potential impaired ventilation–perfusion mismatching Increased risk of PGD Less pulmonary functional reserve
Bilateral LTx	Effective amelioration of pulmonary pressure More pulmonary functional reserve Better survival than single LTx More accessible donor lungs More equitable and reasonable allocation of donor lungs	Increased anesthesia, operative, and bypass time
HLTx	Effective amelioration of pulmonary pressure More pulmonary functional reserve Indication for severe right ventricle and left ventricle dysfunction Indication for Eisenmenger syndrome complicating complex CHD, failed CHD repair, and uncorrectable CHD	Less surgical proportion at all transplant institutions Less accessible donor lungs Potential increased waitlist time and waitlist mortality Rejection risk (heart)

LTx, lung transplantation; HLTx, heart and lung transplantation; CHD, congenital heart disease; PGD, primary graft dysfunction.

## Data Availability

The data are available on request from the corresponding authors.
